# Chemically Functionalized Water-Soluble Single-Walled Carbon Nanotubes Obstruct Vesicular/Plasmalemmal Recycling in Astrocytes Down-Stream of Calcium Ions

**DOI:** 10.3390/cells9071597

**Published:** 2020-07-01

**Authors:** Manoj K. Gottipati, Elena Bekyarova, Robert C. Haddon, Vladimir Parpura

**Affiliations:** 1 Departments of Neurobiology and Biomedical Engineering, University of Alabama at Birmingham, Birmingham, AL 35294, USA; manojgottipati@uab.edu; 2 Department of Biomedical Engineering and Center for Biotechnology and Interdisciplinary Studies, Rensselaer Polytechnic Institute, Troy, NY 12180, USA; 3 Department of Neuroscience, Center for Brain and Spinal Cord Repair and Wexner Medical Center, The Ohio State University, Columbus, OH 43210, USA; 4 Departments of Chemistry and Chemical and Environmental Engineering and Center for Nanoscale Science and Engineering, University of California, Riverside, CA 92521, USA; elenab@ucr.edu; 5 Carbon Solutions, Inc., Riverside, CA 92507, USA

**Keywords:** carbon nanotubes, astrocytes, Ca^2+^ dynamics, glutamate release, membrane recycling

## Abstract

We used single-walled carbon nanotubes chemically functionalized with polyethylene glycol (SWCNT-PEG) to assess the effects of this nanomaterial on astrocytic endocytosis and exocytosis. We observed that the SWCNT-PEG do not affect the adenosine triphosphate (ATP)-evoked Ca^2+^ elevations in astrocytes but significantly reduce the Ca^2+^-dependent glutamate release. There was a significant decrease in the endocytic load of the recycling dye during constitutive and ATP-evoked recycling. Furthermore, SWCNT-PEG hampered ATP-evoked exocytotic release of the loaded recycling dye. Thus, by functionally obstructing evoked vesicular recycling, SWCNT-PEG reduced glutamate release from astrocytes via regulated exocytosis. These effects implicate SWCNT-PEG as a modulator of Ca^2+^-dependent exocytosis in astrocytes downstream of Ca^2+^, likely at the level of vesicle fusion with/pinching off the plasma membrane.

## 1. Introduction

Single-walled carbon nanotubes (SWCNTs) have been considered candidates for applications in biotechnology and biomedicine [1,2], and in particular for neural applications [3,4]. When chemically functionalized with polyethylene glycol (PEG), SWCNTs render water solubility [5]. These colloidal solutes, SWCNT-PEG, have been used to modulate the morpho-functional properties of two main neural cell types, neurons [6] and astrocytes [7], in culture. SWCNT-PEG caused an increase in the neurite outgrowth of selected neurites in vitro and affected the neuronal Ca^2+^ dynamics by reducing the depolarization-dependent influx of Ca^2+^ from the extracellular space [6]. Furthermore, in neurons exposed to SWCNT-PEG, the evoked exocytotic incorporation of vesicles into the plasma membrane was not sufficiently balanced by endocytotic retrieval, a possible mechanism underlying an increase in the neurite outgrowth [8]. In vivo application of the SWCNT-PEG solute resulted in an increase in axonal regeneration and a modest functional locomotor recovery without altering reactive astrogliosis in an acute spinal cord injury rat model [9]. A detailed analysis of astrocytes in cell culture showed an increase in the immunoreactivity of the astrocyte-specific intermediate filament, glial fibrillary acidic protein (GFAP), in the presence of SWCNT-PEG colloidal solute [7]. Astrocytes also became larger and less round; these effects likely report on increased maturity of astrocytes and were GFAP-dependent [10]. SWCNT-PEG solute also caused an increase in the uptake of extracellular glutamate [11] thus affecting glutamate homeostasis in astrocytes. These effects of SWCNT-PEG solute on astrocytes are suggestive of possible actions of CNTs at the plasma membrane, similar to those seen in neurons and likely affecting the membrane recycling and/or intracellular Ca^2+^ dynamics. Astrocytes not only uptake glutamate, but also have the ability to release glutamate into the extracellular space in a Ca^2+^-dependent manner [12] through the process of vesicular exocytosis [13]. Astrocytic glutamate release via regulated exocytosis of glutamate-containing vesicles may play an epileptogenic role in the initiation of epileptic seizures [14,15] and contributes to neuronal excitotoxicity in status epilepticus [16]. Astrocytes isolated from a mouse model of Huntington’s disease showed enhanced excitotoxic glutamate release, which can contribute to glutamate-mediated neuronal excitotoxicity in this disorder [17]. Here, we assessed if SWCNT-PEG affect the Ca^2+^ dynamics in astrocytes and the subsequent glutamate release using genetically encoded intracellular Ca^2+^ along with extracellular glutamate sensors, respectively. Additionally, using a recycling dye, we assessed if SWCNT-PEG affect the vesicular/plasma membrane recycling in astrocytes.

Neurons show electrical excitability, which drives Ca^2+^ dynamics due to the influx of Ca^2+^ from the extracellular space through plasmalemmal voltage-gated Ca^2+^ channels. However, astrocytes are not electrically excitable and do not possess high enough density of voltage-gated channels [18]. Rather, astrocytes display Ca^2+^ excitability that is mainly driven by the activation of plasmalemmal metabotropic receptors [19]. The activity of these receptors and their downstream signaling can lead to increases in cytosolic Ca^2+^ due to the release of this cation from the intracellular store of the smooth endoplasmic reticulum (ER). The store is replenished by store-specific Ca^2+^-ATPase that draws on Ca^2+^ entry from the cytosol and ultimately from the extracellular space, in particular through transient receptor potential canonical 1 protein (TRPC1)-containing channels in astrocytes [20]. This signaling cascade can result in gliotransmission, i.e., Ca^2+^-dependent release of various gliotransmitters, including glutamate, into the extracellular space via vesicular exocytosis [21,22]. 

Adenosine triphosphate (ATP) as a transmitter can activate astrocytic metabotropic receptors resulting in Ca^2+^ dynamics, which critically rely on the supply of Ca^2+^ from the ER store [23], and consequential vesicular release of glutamate into the extracellular space [13,20,24,25]. We observed that the SWCNT-PEG do not affect the ATP-evoked Ca^2+^ elevations in astrocytes but significantly reduce the Ca^2+^-dependent glutamate release, implicating the modulation of this process downstream of Ca^2+^, likely at the level of vesicle fusion with/pinching off the plasma membrane. Indeed, we observed a significant decrease in the total and vesicular load of the recycling dye during constitutive recycling. Furthermore, in pulse and chase experiments, ATP-stimulated recycling (pulse) in the presence of CNTs also resulted in a decrease in the total and vesicular load of the recycling dye and SWCNT-PEG hampered evoked exocytotic release of the loaded recycling dye in the follow-up, second ATP stimulation (pulse). Our findings implicate that SWCNT-PEG cause a decrease in constitutive and evoked endocytic load, offering a possible explanation for our previously reported morphological enlargement of astrocytes [7,10] and increased glutamate uptake, presumably due to prolonged retention of glutamate transporters at the plasma membrane [11]. Furthermore, SWCNT-PEG functionally obstructed evoked vesicular recycling thereby reducing glutamate release from astrocytes via regulated exocytosis.

## 2. Materials and Methods

### 2.1. Cell Culture

All animal procedures were in strict accordance with the National Institutes of Health Guide for Care and Use of Laboratory Animals and were approved by the University of Alabama at Birmingham Institutional Animal Care and Use Committee (Animal project number IACUC-09230 approved on 08/19/2019). Astrocytes isolated from the visual cortices of 0−2-day-old C57BL/6 mice were purified, maintained in cell culture and plated onto polyethyleneimine (PEI)-coated glass coverslips as we previously described in detail elsewhere [7,26]. SWCNT-PEG solute was synthetized and characterized as we previously described elsewhere [7]. The batch of SWCNT-PEG solute used in this study contained 72.3 weight percent (wt %) of the SWCNT backbone, 22.6 wt % of the functional group PEG (PEG 600; MW range 573–630) and 5.1 wt % of metal impurities (nickel and yttrium in ~ 4:1 weight ratio) [11]. In the experiments using the functional group PEG alone, as a control for 5 μg/mL of SWCNT-PEG solute-treated group, PEG was added to the cells at 1 μg/mL, i.e., at the concentration corresponding to 20 wt % of the SWCNT-PEG solute. 

### 2.2. Transfection

Control and SWCNT-PEG solute-treated astrocytes were transfected 1 day after plating with one of the following plasmids encoding: (i) RCaMP1h, kindly provided by Dr. Loren Looger, (Howard Hughes Medical Institute, Ashburn, VA, USA) [27,28], (ii) iGluSnFR, purchased from Addgene (Watertown, MA, USA; Cat No. 41732) [29], or iii) Lck_1-26_-EGFP, EGFP tagged with the first 26 amino acids of Lck at its N-terminus, kindly provided by Dr. Steven H. Green (University of Iowa, Iowa City, IA, USA) [30,31]. Each dish containing 4 coverslips with cells received 0.5 μg of the plasmid DNA and 1 μL of TransIT-293 transfection agent (Mirus Bio, Madison, WI, USA), premixed as per manufacturer’s instructions. After 4 h, the dishes were washed with Hank’s balanced salt solution (HBSS) and replaced with fresh cell culture media with or without the addition of 5 µg/mL SWCNT-PEG or 1 μg/mL PEG. The cell culture medium contained α-minimum essential medium (without phenol red; Invitrogen) supplemented with fetal bovine serum (10%, Hyclone), sodium bicarbonate (14 mM), sodium pyruvate (1 mM), D-glucose (20 mM), L-glutamine (2 mM), penicillin (100 IU/mL) and streptomycin (100 μg/mL) (pH 7.35). All the dishes were returned to a 37 °C 95% air/5% CO_2_ incubator for 3 days until used in experiments.

### 2.3. Imaging

All imaging experiments were done at room temperature (22−25 °C) using a light microscope (Nikon TE300) equipped with bright field and differential interference contrast (DIC; halogen lamp, 100 W), along with wide-field epifluorescence illumination (xenon arc lamp, 100 W). The images were acquired using a CoolSNAP-HQ^2^ cooled, charge coupled device camera (Photometrics, Tucson, AZ) driven by V++ imaging software (Digital Optics, Auckland, New Zealand). Coverslips containing cultured astrocytes were mounted onto an imaging chamber filled with an aqueous external solution containing (in mM): NaCl (140), KCl (5), CaCl_2_ (2), MgCl_2_ (2), D-glucose (5), and Hepes (10) (pH 7.4). All the raw images had pixel intensities without saturation and within the dynamic range of the camera (0−16,383). 

### 2.4. Ca^2+^ Dynamics

To study the ATP-induced Ca^2+^ dynamics, time-lapse imaging was done on the astrocytes expressing RCaMP1h, 3 days post-transfection (total of 4 days in culture and SWCNT-PEG/PEG treatment where applicable), and its fluorescence was visualized and imaged using a standard tetramethylrhodamine isothiocyanate (TRITC) filter set. Images were acquired using a 60× Plan Apo objective (Nikon; numerical aperture, 1.4) and the microscope described above. After a cell of interest was identified based on its fluorescence, two subsequent time-lapse epochs were acquired to monitor the intracellular Ca^2+^ levels. During the 1^st^ epoch (260 s, 1 frame/5 s), we replaced the external solution with external solution containing ATP (100 µM) after the 6^th^ frame. For a subset of the experiments, the external solution was supplemented with cyclopiazonic acid (CPA; 20 µM, Sigma) for the entire course of the experiment (Supplementary material and Figure S1). Following the completion of this epoch, the ATP-containing external solution was replaced with external solution containing 4-Bromo-A23187 (4-Br; 20 µM, Molecular Probes) and the 2^nd^ epoch was acquired (150 s, 1 frame/15 s). The background (area of the coverslip containing no cells) subtracted RCaMP1h fluorescence intensity was expressed as dF/F_0_ (%), where dF represents the change in fluorescence, while F_0_ represents the baseline fluorescence of the cell before ATP stimulation (an average of the first 6 frames in the 1^st^ epoch). The external solution in both the epochs also contained 5 µg/mL SWCNT-PEG for all the cells treated with SWCNT-PEG. 

### 2.5. Glutamate Release 

To study the ATP-induced glutamate release and plasma membrane dynamics, time-lapse imaging was done on astrocytes transfected with plasmids encoding iGluSnFR or Lck_1-26_-EGFP (Supplementary material and Figure S2), respectively, 3 days post-transfection (total of 4 days in culture and SWCNT-PEG/PEG treatment where applicable), and their fluorescence was visualized and imaged using a standard fluorescein isothiocyanate (FITC) filter set. To monitor the extracellular glutamate levels, imaging was done as described above except that the external solution in the 2^nd^ epoch contained exogenous glutamate (100 µM) instead of 4-Br. For a subset of the experiments, the external solution in the 1^st^ epoch was replaced with external solution lacking ATP (Supplementary material and Figure S3). 

### 2.6. Plasma Membrane Recycling 

To assess plasma membrane recycling, cultured astrocytes were loaded with *N*-(3-triethylammoniumpropyl)-4-(6-(4-(diethylamino)phenyl)hexatrienyl)pyridinium dibromide (FM4-64; 10 µM, 5 min, Molecular Probes) without and with the addition of ATP (100 µM) for constitutive and ATP-stimulated recycling, respectively, 4 days post-plating. FM4-64 was visualized using a standard TRITC filter set and time-lapse imaging was done using a 20× Plan Fluor objective (Nikon; numerical aperture, 0.5) in two epochs. During the 1^st^ epoch the baseline fluorescence was acquired in external solution (50 s, 1 frame/10 s), which was replaced with external solution containing FM4-64 + ATP. After washing off the excess FM4-64, the 2^nd^ epoch was acquired in external solution (500 s, 1 frame/10 s). The data was reported as the background (area of the coverslip containing no cells) subtracted FM4-64 fluorescence intensity, dF. The baseline auto-fluorescence of astrocytes before the addition of FM4-64 was used as F_0_. To obtain the outlines of the cells, prior to FM4-64 loading, astrocytes were loaded with β-Ala-Lys-Nε-AMCA (20 μM at 37°C for 2 h in cell culture medium; AMCA, 7-amino-4-methylcoumarin-3-acetic acid) and imaged using a standard 4′,6-diamidino-2-phenylindole (DAPI) filter set. To study the ATP-stimulated exocytosis of FM4-64, the external solution in the 2^nd^ epoch was replaced with external solution containing ATP (100 µM) after the 50^th^ frame and the imaging was continued (300 s, 1 frame/10 s). The external solution in all the experiments also contained 5 µg/mL SWCNT-PEG or 1 µg/mL PEG for the cells with respective treatments. 

### 2.7. Statistics 

**Figure 1:**
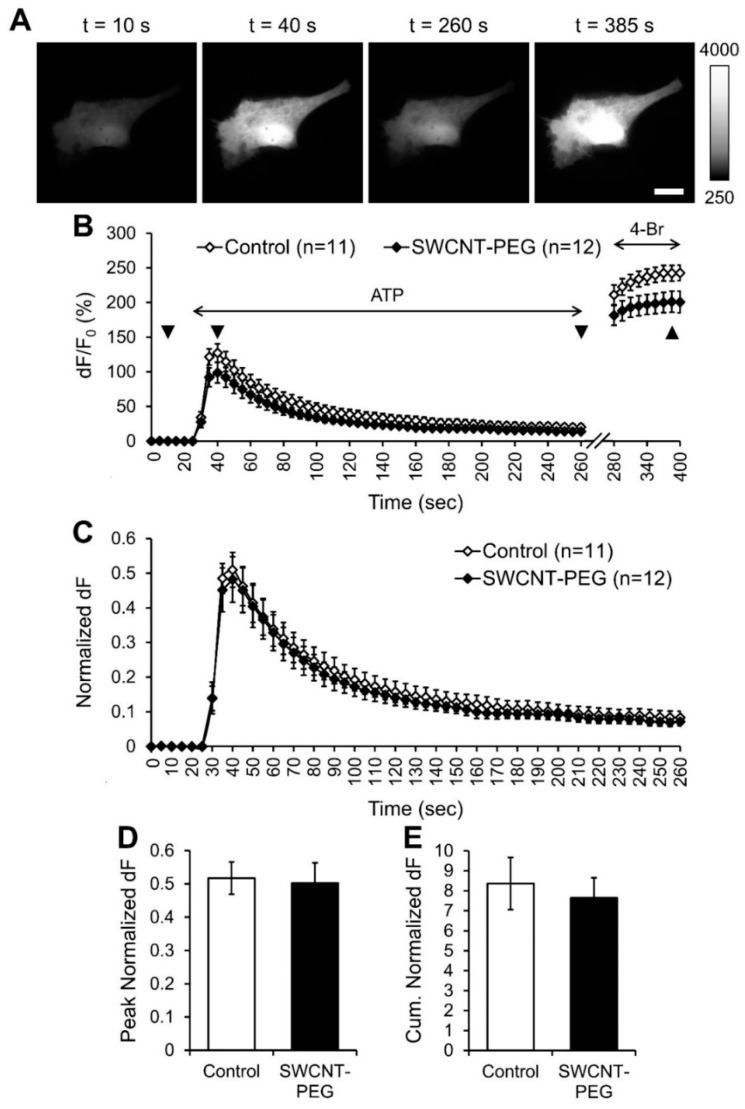
Single-walled carbon nanotubes chemically functionalized with polyethylene glycol (SWCNT-PEG) do not affect the ATP-induced intracellular Ca^2+^ elevations in cultured mouse cortical astrocytes. (**A**) Individual frames from the time-lapse imaging of RCaMP1h (intracellular Ca^2+^ sensor) in a control astrocyte showing the changes in RCaMP1h fluorescence at selected time points (indicated with triangles in (**B**)). Scale bar, 20 µm. Gray scale is a linear representation of the fluorescence intensities of the pixels in the images, expressed in fluorescence intensity units (iu). (**B**) Time-lapse imaging of RCaMP1h fluorescence, reporting on the average intracellular Ca^2+^ levels in astrocytes in the absence and the presence of SWCNT-PEG solute (5 µg/mL). ATP (100 µM) was bath applied to elicit an increase in intracellular Ca^2+^ levels and the Ca^2+^ ionophore 4-Bromo-A23187 (4-Br; 20 µM) was bath applied to elicit maximal Ca^2+^ response in astrocytes. The horizontal double-headed arrows indicate the times of addition of ATP and 4-Br containing external solutions. Changes in RCaMP1h fluorescence are expressed as dF/F_0_ (%) after background subtraction. Number of astrocytes studied in each condition is shown in parentheses. (**C**) Changes in the RCaMP1h fluorescence of astrocytes shown in B, normalized to their maximal Ca^2+^ response after the application of 4-Br, expressed as the normalized change in RCaMP1h fluorescence, dF. Traces in (**B**) and (**C**) show means + SEMs. (**D**,**E**) Summary graphs showing the average peak (**D**) and cumulative (**E**) normalized dF with SEMs.

**Figure 2:**
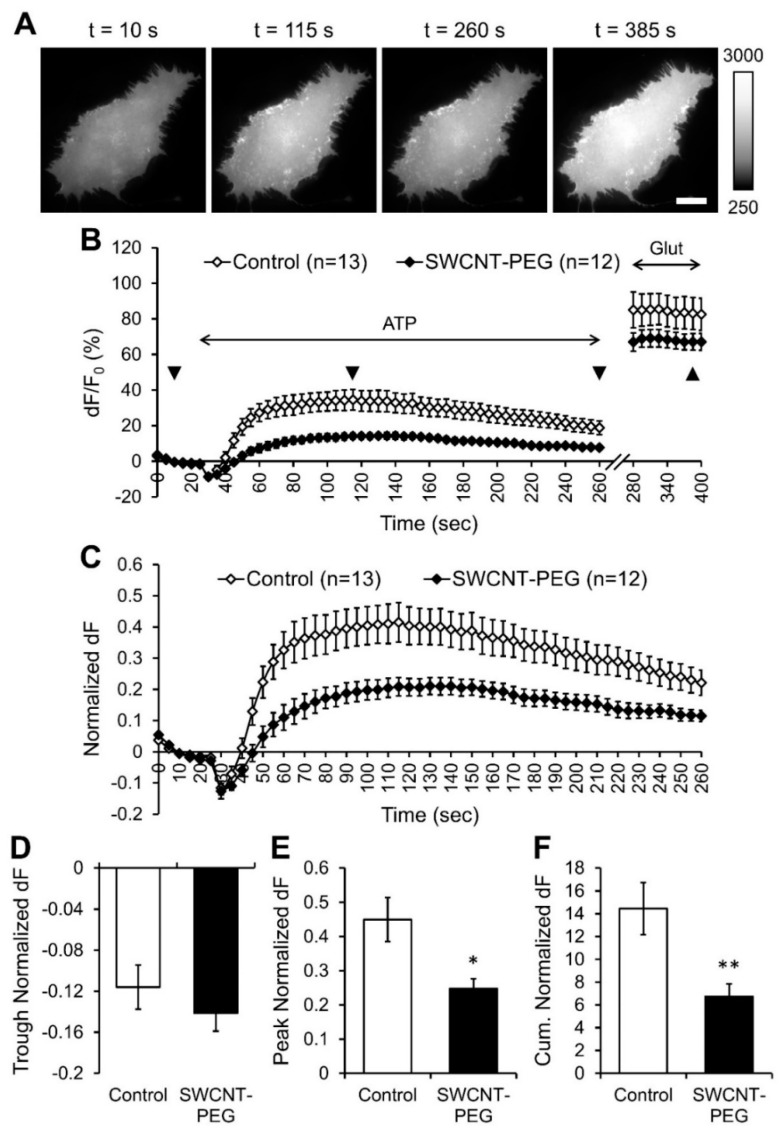
SWCNT-PEG solute inhibits the ATP-induced glutamate release from cultured mouse cortical astrocytes. (**A**) Individual frames from the time-lapse imaging of iGluSnFR (extracellular glutamate sensor) in a control astrocyte showing the changes in iGluSnFR fluorescence at selected time points (indicated with triangles in (**B**)). Scale bar, 20 µm. Gray scale is a linear representation of the fluorescence intensities of the pixels in the images, expressed in fluorescence intensity units (iu). (**B**) Time-lapse imaging of iGluSnFR fluorescence, reporting on the average extracellular glutamate levels at the plasma membrane of astrocytes in the absence and the presence of SWCNT-PEG solute (5 µg/mL). ATP (100 µM) was bath applied to stimulate Ca^2+^-dependent exocytotic glutamate release from astrocytes. Exogenous glutamate (Glut; 100 µM) was bath applied to saturate the iGluSnFR fluorescence at the plasma membranes of astrocytes. The horizontal double-headed arrows indicate the times of addition of ATP and glutamate containing external solutions. Changes in iGluSnFR fluorescence are expressed as dF/F_0_ (%) after background subtraction. Number of astrocytes studied in each condition is shown in parentheses. (**C**) Changes in the iGluSnFR fluorescence of astrocytes shown in B, normalized to their saturated glutamate response after the application of glutamate, expressed as the normalized change in iGluSnFR fluorescence, dF. Traces in B and C show means + SEMs. (**D**–**F**) Summary graphs showing the average trough (**D**), peak (**E**) and cumulative (**F**) normalized dF with SEMs. Asterisks indicate a statistical difference compared to the control. Student’s *t*-test (pooled variances); * *p* < 0.05, ** *p* < 0.01.

**Figure 3:**
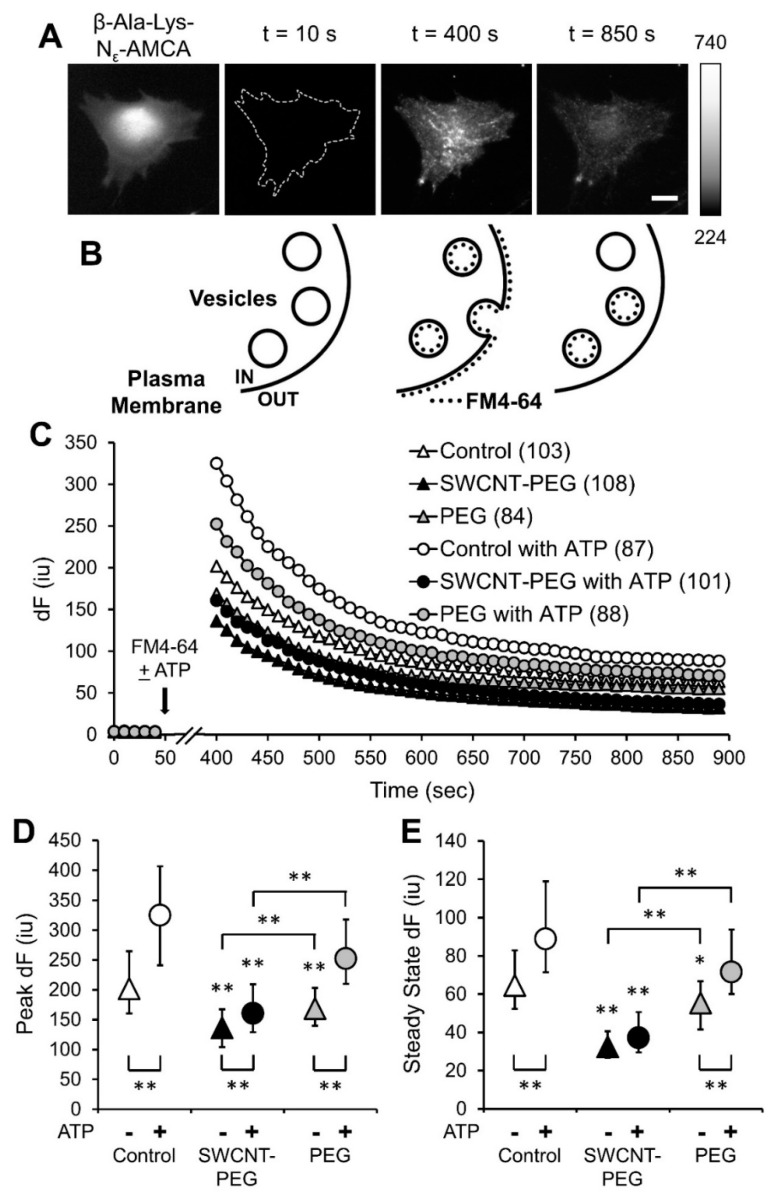
SWCNT-PEG solute inhibits the endocytotic load of the recycling dye FM4-64 during constitutive and ATP-stimulated recycling in cultured mouse cortical astrocytes. (**A**) Images (from left to right) showing a control astrocyte loaded with β-Ala-Lys-Nε-AMCA (DAPI filter set) and three subsequent individual frames from the time-lapse imaging showing the changes in FM4-64 fluorescence intensity (TRITC filter set) before the addition of FM4-64 (t = 10 s), immediately after the addition of FM4-64, (peak; t = 400 s), and at steady state (t = 850 s). Scale bar, 50 µm. The t = 10 s image shows astrocyte autofluorescence (TRITC filter set) with the dotted outline representing the cell area traced based on the corresponding β-Ala-Lys-Nε-AMCA image (left). Gray scale is a linear representation of the fluorescence intensities of the pixels in the images, expressed in fluorescence intensity units (iu). (**B**) Schematics showing FM4-64 labeling and membrane recycling in astrocytes before dye application, after dye application and at steady state after the dye washout, respectively. (**C**) Time-lapse imaging of FM4-64 fluorescence in the absence and the presence of SWCNT-PEG solute (5 µg/mL) or PEG (1 µg/mL). The arrow indicates the beginning of the 5 min FM4-64 (10 µM) application without or with ATP (100 µM) to study constitutive (triangles) or ATP-stimulated (circles) recycling, respectively (broken abscissa). Number of astrocytes studied in each condition is shown in parentheses. Traces show the background subtracted FM4-64 fluorescence (dF) reported as medians. (**D**,**E**) Summary graphs showing the median peak (**D**) and steady state (**E**) of FM-4-64 dF with interquartile range. Asterisks indicate a statistical difference compared to the corresponding control group. Other differences are marked by the brackets. KWA followed by Dunn’s test; * *p* < 0.05, ** *p* < 0.01.

**Figure 4:**
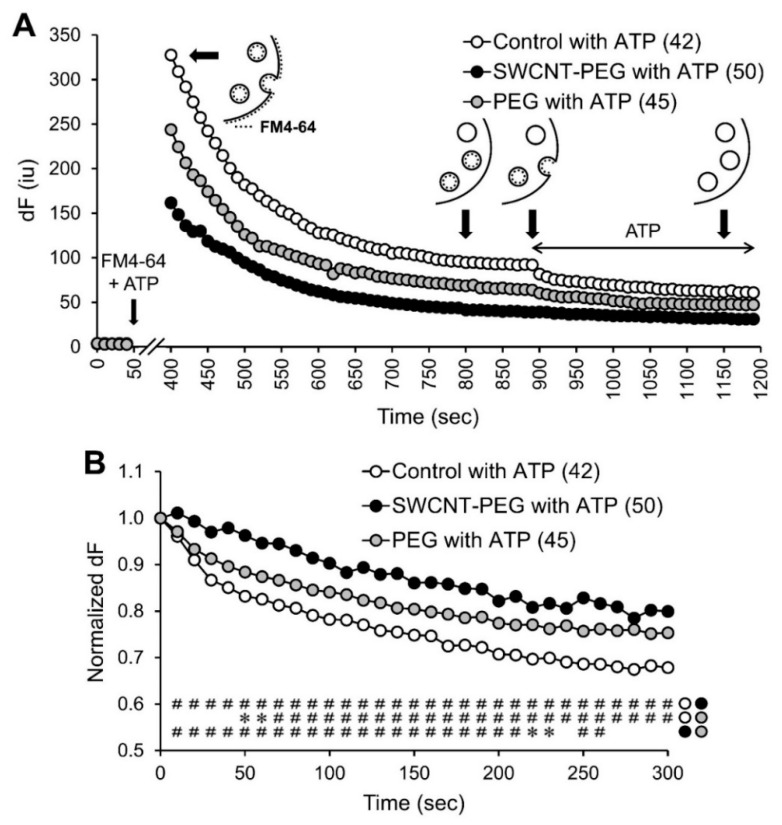
SWCNT-PEG solute inhibits the ATP-stimulated exocytosis (chase) of the FM4-64 pre-loaded in exocytosis vesicles by ATP-stimulation (pulse) of cultured mouse cortical astrocytes. (**A**) Time-lapse imaging of FM4-64 fluorescence for astrocyte groups stimulated with ATP once and washed as described in Figure 3 (pulse), followed by second ATP (100 µM) application to stimulate the exocytosis of FM4-64 loaded in the vesicles (chase), the time of addition of which is indicated by the horizontal double-headed arrow. Other annotations as in Figure 3. Number of astrocytes studied in each condition is shown in parentheses and represents a fraction of the astrocytes already reported in Figure 3 but here stimulated again with ATP for the second time. The schematics show FM4-64 labeling and membrane recycling in astrocytes after dye application, at steady state after the dye washout, ATP-stimulated exocytosis and at steady state after ATP-stimulated dye washout, respectively (left to right), the time points of which are marked by the bold arrows. (**B**) FM4-64 fluorescence normalized to the steady-state level just prior to the second application of ATP. Traces in (**A**) and (**B**) show medians. Asterisks and pound signs indicate a statistical difference between the groups indicated on the right at the specific time points. KWA followed by Dunn’s test; * *p* < 0.05, # *p* < 0.01.

Statistical analysis was done using GraphPad Prism 8 Statistical Software (GraphPad Software, San Diego, CA) and SAS Software, version 9.4, of the SAS software for Windows (SAS Institute Inc., Cary, NC). The number of subjects required for individual set of experiments was estimated using power analysis (set at 80% and α = 0.05). The summary graphs are reported as means ± standard errors of means (SEMs) or medians with the interquartile range (IQR). Traces in Figure 1 and Figure 2 are reported as means ± SEM, while the traces in Figure 3 and Figure 4 are reported as medians without IQRs, for simplicity. All the experiments contain cells originating from at least three independent experimental runs/culture preparations. Student’s t-test (pooled variances) was used for the experiments comparing the two independent groups conforming to normality based on Shapiro-Wilk test for normality (Figure 1, Figure 2, Figures S1 and S3). For data sets containing groups that deviated from normality, nonparametric statistics were used, with multiple independent groups analyzed using Kruskal−Wallis one-way ANOVA (KWA) followed by Dunn’s test (Figure 3 and Figure 4). The significance was established at *p* < 0.05.

## 3. Results

In the present study, we assessed the effect of SWCNT-PEG colloidal solute on the Ca^2+^ dynamics and the consequential glutamate release from cortical astrocytes in response to ATP stimulation. We monitored the levels of intracellular Ca^2+^ and extracellular glutamate using the genetically encoded intracellular Ca^2+^ indicator, RCaMP1h [27] and the genetically encoded plasma membrane anchored extracellular glutamate sensor, iGluSnFR [29], respectively. We plated astrocytes onto polyethyleneimine (PEI)-coated glass coverslips in the absence and the presence of 5 µg/mL SWCNT-PEG and after 1 day in culture, transfected the cells with one of the two plasmids, RCaMP1h or iGluSnFR. Three days post-transfection and the continued treatment of a subset of cells with SWCNT-PEG (total of 4 days of SWCNT-PEG), the cells were visualized and imaged using a fluorescence microscope and standard TRITC (for RCaMP1h) or FITC (for iGuSnFR) filter sets. Time-lapse imaging was done to study astrocytic changes in intracellular Ca^2+^ levels, [Ca^2+^]_i_ (Figure 1A) and extracellular glutamate levels, [Glut]_e_ (Figure 2A). 

### 3.1. SWCNT-PEG does not Affect ATP-evoked Ca^2+^ Dynamics in Astrocytes 

Astrocytes were exposed to ATP (100 µM) to induce [Ca^2+^]_i_ elevations in these cells via the ER Ca^2+^ stores (Supplementary material and Figure S1). This was followed by the application of the Ca^2+^ ionophore 4-Bromo-A23187 (4-Br; 20 µM) that, upon incorporation into the plasma membrane, presents a conduit for Ca^2+^ entry from the extracellular space to elicit a maximal [Ca^2+^]_i_ response [32,33] (Figure 1A,B). As expected, bath application of ATP caused a steep transient increase in the [Ca^2+^]_i_, seen as an increase in RCaMP1h fluorescence, in all the control cells (n = 11) studied (Figure 1B). The subsequent bath application of 4-Br elicited maximal [Ca^2+^]_i_ responses as per RCaMP1h saturation (~ 10 µM; ref. [27]) due to the entry of Ca^2+^ from the extracellular space (2 mM Ca^2+^ in the external solution) into the cytosol (Figure 1B). All the data were background subtracted and expressed as the percentage change (dF/F_0_) in RCaMP1h fluorescence compared to the baseline RCaMP1h fluorescence before ATP stimulation (F_0_; Figure 1B). The cells treated with and imaged in presence of SWCNT-PEG (n = 12) showed similar ATP-evoked increase in the [Ca^2+^]_i_ as control cells. Since the maximal 4-Br evoked [Ca^2+^]_i_ responses were significantly different between the CNT-treated and untreated groups (Student’s t-test, *p* = 0.02), we normalized the changes in the [Ca^2+^]_i_ for each of the cells studied to their respective maximum [Ca^2+^]_i_ responses (Figure 1C). We found that the SWCNT-PEG solute didn’t cause any significant differences in the peak (Figure 1D) or the cumulative (Figure 1E) normalized dF compared to the control implying that the SWCNT-PEG solute doesn’t affect the ATP-induced Ca^2+^ dynamics in astrocytes.

### 3.2. SWCNT-PEG Hampers ATP-evoked Glutamate Release from Astrocytes

Next, we assessed the effect of SWCNT-PEG solute on the ATP-induced glutamate release from astrocytes. After baseline acquisition of iGluSnFR fluorescence, the cells were stimulated with ATP (bath application, 100 µM) and the resultant release of glutamate into the extracellular space was recorded as the change in iGluSnFR fluorescence (Figure 2A,B). Following ATP stimulation, the cells were also challenged with exogenously added glutamate (Glut; bath application, 100 µM) to obtain the maximum/saturation fluorescence of iGluSnFR [29] (Figure 2A,B). All the data were background subtracted and expressed as the percentage change (dF/F_0_) in iGluSnFR fluorescence compared to the baseline iGluSnFR fluorescence before ATP stimulation (F_0_; Figure 2B). We found that all the control cells (*n* = 13) studied showed a rapid transient decrease, a trough, in iGluSnFR fluorescence after the addition of ATP, indicative of a decrease in extracellular glutamate level (Figure 2B), followed by a slower transient increase in fluorescence, indicative of an increase in extracellular glutamate level. While the trough represents a temporal dilution of glutamate in the extracellular milieu as a result of the bath exchange that took place when ATP was applied (Supplementary Material and Figures S2 and S3), the remaining signal represents a typical time course of evoked astrocytic glutamate release [24,25]. The cells treated with and imaged in presence of SWCNT-PEG (*n* = 12) also showed a similar time course in the change of iGluSnFR fluorescence (Figure 2B). However, the magnitude of the increase in fluorescence was much lower than that in the control cells. Similar to the 4-Br evoked maximal [Ca^2+^]_i_ response, the saturated iGluSnFR fluorescence was also significantly different between the CNT-treated and untreated groups (Student’s *t*-test, *p* < 0.01). Thus, we normalized the changes in the extracellular glutamate levels for each of the cells studied to their respective maximum iGluSnFR fluorescence (Figure 2C). We found that the SWCNT-PEG solute did not cause any significant difference in the trough observed immediately upon ATP application (Figure 2D), as expected from a perfusion artifact. Unexpectedly, however, SWCNT-PEG caused a significant reduction in the peak (Figure 2E) and cumulative (Figure 2F) normalized dF compared to the control cells implying that the SWCNT-PEG solute hampers the ATP-induced glutamate release from astrocytes without affecting Ca^2+^ dynamics in astrocytes. We showed previously that the functional group PEG itself does not affect glutamate homeostasis while SWCNT-PEG increased the glutamate uptake in astrocytes (Figure 1B of Reference [11]). Hence, the effect that we observed here is likely due to the SWCNT backbone and not the PEG functionalization group.

There is a strict relationship between cytosolic Ca^2+^ increase and the amount of exocytotically released glutamate from astrocytes [32]. Under certain conditions, this relationship can be modulated down stream of Ca^2+^ signal [17,24,34,35]. Thus, the disparity between the Ca^2+^ and glutamate dynamics in the presence of SWCNT-PEG solute we observed in the present work (compare Figure 1C–E to Figure 2C and Figure 2E,F, respectively) represents a negative modulation down stream of Ca^2+^ signal. In part, this effect could be explained by the fact that SWCNT-PEG augmented glutamate uptake by astrocytes, as we reported elsewhere [11]. However, the reported 26% increase in glutamate uptake (Figure 1B of Reference [11]) may only explain about half of the 53% diminution of glutamate release seen here (Figure 2F). As SWCNT-PEG can tamper with vesicular recycling in neurons [8], it is possible that the negative modulation of the relationship between astrocytic cytosolic Ca^2+^ and released glutamate could be due to an aberration in astrocytic vesicular trafficking and/or membrane recycling, which we explored next. 

### 3.3. SWCNT-PEG Inhibits Constitutive and ATP-stimulated Membrane Recycling in Astrocytes

To assess if SWCNT-PEG solute interferes with plasma membrane-vesicular recycling in astrocytes, we used a recycling dye, FM4-64 [36,37]. Since FM4-64 does not passively diffuse across cell membranes, it is taken up by endocytosis. After 4 days in culture, astrocytes in the absence and the presence of 5 µg/mL SWCNT-PEG were preloaded with a dipeptide β-Ala-Lys conjugated to 7-amino-4-methylcoumarin-3-acetic acid (AMCA) (20 μM) [24]; this peptide is specifically taken up by the astrocytes via pepT2 peptide transporter [38] into their cytosol. We imaged dipeptide loaded astrocytes using a standard DAPI filter set to get the proper outlines of the cells (Figure 3A). This outline aided cell autofluorescence assessment in the subsequent time-lapse imaging, which was done to study the changes in FM4-64 fluorescence using a fluorescence microscope and a standard TRITC filter set (Figure 3A). After baseline acquisition to measure the autofluorescence level of astrocytes (used as F_0_ after background subtraction) (Figure 3A, t = 10 s and Figure 3B, left), the cells were exposed to FM4-64 (10 μM, 5 min) to monitor constitutive vesicular recycling which occurs in unstimulated cells. The dynamics in the FM4-64 fluorescence overtime was recorded and reported as the background subtracted FM4-64 fluorescence intensity, dF (Figure 3C). The peak FM4-64 fluorescence measured 5 min after FM4-64 exposure reported on the extent of total FM4-64 cellular load (plasma membrane and vesicles) (Figure 3A, t = 400 s and Figure 3B, middle), while the steady state FM4-64 fluorescence after extensive rinsing reported on the vesicular load since plasma membrane bound FM4-64 re-partitioned in the external solution and washed away (Figure 3A, t = 850 s and Figure 3B, right). In unstimulated control cells, there was an expected exponential decrease (R^2^ = 0.8774) in FM4-64 fluorescence with decay time constant, i.e., time to reach dF at maximum/exponent e (~37% maximum fluorescence), of 700 s. The unstimulated cells treated with SWCNT-PEG solute also showed an exponential decrease (R^2^ = 0.9128) in FM4-64 fluorescence but substantially shorter decay time constant of 600 s, along with a significant decrease in the peak (Figure 3D) and steady state dF (Figure 3E) of FM4-64 fluorescence. When combined, these data imply that SWCNT-PEG cause a decrease in constitutive endocytosis which could represent an underlying mechanism for our previously reported increase in astrocytic size [7,10] and for an increase of glutamate uptake by astrocytes [11]; the latter mediated by an increase in the amount of L-glutamate/L-aspartate transporter (GLAST/ EAAT1) at the plasma membrane. These findings also imply an imbalance between constitutive exocytosis and endocytosis processes.

Since vesicular recycling in astrocytes can be evoked in a Ca^2+^-dependent manner, we studied the effect of SWCNT-PEG on ATP-stimulated vesicular recycling by supplementing the FM4-64 containing external solution with ATP (100 µM) and repeating the experiment described above. As expected, the control cells showed a significant increase in the peak and steady state FM4-64 fluorescence in the presence of ATP when compared to unstimulated astrocytes (Figure 3D,E); there was an exponential decrease (R^2^ = 0.8711) in FM4-64 fluorescence with a decay time constant of 620 s. The cells treated with SWCNT-PEG solute and stimulated with ATP also showed an exponential decrease (R^2^ = 0.927) in FM4-64 fluorescence with a comparable decay time constant of 600 s; there was a significant increase only in the peak but not the steady state FM4-64 fluorescence when compared to unstimulated SWCNT-PEG-treated cells (Figure 3D,E). In addition, the ATP-stimulated peak and steady state FM4-64 fluorescence were significantly lower in the cells treated with SWCNT-PEG solute compared to the corresponding control cells (Figure 3D,E). 

We further addressed the possibility that the observed effects might be due to the PEG functional group by treating a subset of the cells with PEG (1 µg/mL; the concentration corresponding to 20 wt % of SWCNT-PEG) and repeating the experiments described above. We found that the unstimulated cells treated with PEG solute showed a significant decrease in the peak (Figure 3D) and steady state (Figure 3E) FM4-64 fluorescence at rest when compared to controls cells, but these values were significantly higher than those observed in the SWCNT-PEG-treated astrocytes. These findings imply that PEG itself could partially affect constitutive recycling in astrocytes. On the other hand, in the presence of ATP, PEG-treated astrocytes showed no significant differences in the peak (Figure 3D) and steady state (Figure 3E) FM4-64 fluorescence compared to the control stimulated astrocytes. These fluorescence values were also significantly higher than the values observed with SWCNT-PEG-treated cells implying that PEG does not affect the ATP-stimulated recycling in astrocytes. Both PEG-treated groups showed exponential decay of FM-64 fluorescence and comparable decay time constants to control groups (for unstimulated cells R^2^ = 0.8343 and 700 s; for ATP-stimulated cells R^2^ = 0.8764 and 620 s, respectively). Taken together, these results imply that the SWCNT-PEG solute affects both the constitutive and ATP-stimulated membrane recycling in astrocytes, with PEG playing a partial role in the constitutive membrane recycling.

### 3.4. SWCNT-PEG Obstructs ATP-evoked Exocytosis in Astrocytes

The total cellular and vesicular load of the recycling dye is the result of a complex interplay between exocytotic and endocytotic processes. To assess if SWCNT-PEG might have an additional effect (to that on endocytosis) on regulated exocytosis, we executed pulse-chase experiments, also referred to as “paired-pulse” experiments [8,39]. The above described loading of astrocytes with FM4-64, utilizing ATP as a stimulus, followed by extensive rinsing (Figure 3C, and replotted in Figure 4A, t = 0–890 s) represent the pulse portion of the experiments. At the steady state level of FM4-64 fluorescence, we executed the “chase” component of the experiments, i.e., we stimulated the astrocytes a second time with external solution containing ATP (100 µM, 5 min) alone and lacking FM4-64 (Figure 4A, t = 900–1190 s). This chase/second stimulus would lead to evoked exocytosis of FM4-64 previously loaded into the vesicles. 

To study the amount and rate of exocytosis, we normalized the decay in FM4-64 fluorescence to the steady state level (t = 890 s) just prior to the application of ATP (Figure 4B). Following ATP stimulation and as expected, we found that the control cells showed an expected exponential decrease (R^2^ = 0.9044) in FM4-64 fluorescence as the ATP application caused exocytosis of the FM4-64 loaded in vesicles. Astrocytes show a very slow time course of regulated exocytosis [25,40,41]. Indeed, we calculated a decay time constant of 888 s based on the fitted equation. The SWCNT-PEG-treated cells, on the other hand, showed an unexpected linear decrease (R^2^ = 0.9484) in FM4-64 fluorescence following ATP stimulation. Because of this lack of an exponential fit and to avoid possible errors related to the asymptotic portion of the FM4-64 curves, we assessed the time domain relevant to the rising phase of the ATP-evoked glutamate (Figure 2C) and reported on the time to destain 10% of the FM4-64 load, i.e., t_90_. The t_90_ in the control astrocytes was 20 s, which was grossly extended (5 times) to 100 s in the SWCNT-PEG group, implying a severe deficit in the evoked exocytosis of the loaded FM4-64 in the astrocytes treated with SWCNT-PEG solute. Interestingly, PEG had some effect on the destaining time course. The PEG-treated cells showed an exponential decrease (R^2^ = 0.9045) in FM4-64 fluorescence following ATP stimulation with initial destaining being only ~50% longer (t_90_ = 30 s) than that of control (Figure 4B), while the late portion of the curve resembled more that of SWCNT-PEG-treated astrocytes (Figure 4B). Taken together, our results show a significant decrease in ATP-evoked (pulse) vesicular load in astrocytes treated with SWCNT-PEG, but not with PEG itself. The ATP-evoked exocytosis (chase) of the FM4-64 loaded in vesicles was obstructed by SWCNT-PEG with both of its components, CNT backbone and PEG, having partial effects on ATP-evoked exocytosis.

## 4. Discussion

The ATP-evoked elevations in astrocytic intracellular Ca^2+^ were unaffected in the presence of SWCNT-PEG (Figure 1). This finding is seemingly at odds with our previous report showing that SWCNT-PEG reduced depolarization-dependent Ca^2+^ influx via plasmalemmal voltage-gated channels from the extracellular space [6]. However, ATP-induced Ca^2+^ dynamics in astrocytes mainly result from the activity of the smooth ER Ca^2+^ store (Supplementary material, Figure S1) and TRPC1-containing channels [20]. This would imply that SWCNT-PEG might have some selective antagonistic effect on a subset of plasmalemmal Ca^2+^ channels. Indeed, it has been reported that SWCNTs blocked a variety of phylogenetically distant voltage-gated K^+^ channels, but not Cl^-^ channels implying selectivity to cation channels. Although it is tempting to speculate that electronic properties of SWCNTs might underlie this selectivity, the work on K^+^ channels suggests that it is the shape (tube) and size (~ 1 nm in diameter) of these materials, but not electrochemical interactions, that governed the inhibition of ion channels [42]. Our water-soluble SWCNTs are of similar diameters [6,7,8,11,26] (also see below). 

Unlike ATP-induced Ca^2+^ dynamics that were preserved when astrocytes were exposed to SWCNT-PEG (Figure 1), the consequential/ Ca^2+^-dependent glutamate release was severely reduced (Figure 2), which implicated the negative modulation of the relationship between cytosolic Ca^2+^ increase and the amount of exocytotically released glutamate from astrocytes. Interestingly, such negative modulation has been initially reported when tampering with astrocytic vesicular proteins [24]. Namely, the exocytotic release of glutamate from astrocytes critically relies on the presence of synaptobrevin 2/VAMP2 that docks vesicles to the plasma membrane, while the storage of glutamate in astrocytic vesicles requires the action of vacuolar-type H^+^-ATPase (V-ATPase) that creates H^+^ gradient used by vesicular glutamate transporters (VGLUTs) delivering glutamate into the vesicular lumen [13]. Pre-treatment of astrocytes with a holoprotein of Tetanus toxin, that cleaves synaptobrevin-2, Bafilomycin A1, a specific inhibitor of V-ATPase, or Rose Bengal, an inhibitor of VGLUTs, all reduced mechanically-induced glutamate release, without affecting Ca^2+^ responses. In addition, Rose Bengal reduced ATP-induced glutamate release, without affecting Ca^2+^ responses [24]. Thus, it is possible that SWCNTs would interact with the lumenal portion of these vesicular proteins (and/or other vesicular proteins, lipids, and/or their glycosylated forms) to affect vesicular recycling and ATP-evoked glutamate release. If so, the type of vesicular fusion, size of vesicles and their fusion pores should matter and so would the geometry of SWCNT-PEG. 

Astrocytic vesicles can exhibit full fusions, where vesicular membrane collapses into the plasma membrane with subsequent emergence of endocytic vesicles by pinching off the plasma membrane, or transient fusions, so called kiss-and-run fusions, where a fusion pore is transiently formed between the vesicular lumen and the extracellular space, vesicle undocks and then locally recycles. When stimulated with ATP, astrocytes displayed more full (on average 64%) than transient (on average 36%) vesicle fusion events [25] with vesicles in live astrocytes measuring ~ 300 nm in diameter [25,43]. When astrocytes are bathed in a saline containing Ca^2+^ at physiological extracellular levels (2 mM; same as here), spontaneous transient fusion pores can be, due to technical limitations, classified into two populations (50% each), one with vesicles with a wider, over 2.6 nm in diameter, pore size and the other with vesicles that have a narrower, on average 0.75 nm in diameter, fusion pore size [44]. As per the geometry of our SWCNT-PEG with lengths ranging from 0.1 to 1.8 μm (mode ~0.3 μm) and diameters between 1 and 7 nm (mode ~3 nm) [26], the vesicular lumen of most of the fusing vesicles in astrocytes would be accessible to SWCNT-PEG. This suggests that only ~18% of vesicles (50% of 35% fusion events) would be inaccessible to SWCNTs in astrocytes due to a mismatch between the fusion pore size and diameter of the SWCNTs. The proportion of inaccessible astrocytic vesicles are likely even smaller, however, as ATP stimulation can dilate the fusion pore [45]. Be that as it may, if the physical insertion of SWCNT-PEG into the pore and their interaction with the vesicular lumen drive a reduction in glutamate release, there would be plenty (82% or more) of vesicles available to interact with SWCNTs and produce a 53% diminution of the glutamate release as we report here. However, if the SWCNT-PEG need to be endocytosed via full and/or kiss-and-run fusions in order to exert the effect on recycling, it is their length that would obstruct such a process. Furthermore, several groups have conjugated CNTs with biological molecules to get them into cells [46,47,48,49] albeit the mechanism of translocation into cells is elusive. Anecdotally, we find very rarely that astrocytes (less than 1 in a thousand) accumulate SWCNT-PEG in the cytoplasm (Figure S4), which would implicate that endocytosis and/or CNT translocation into the cytosol are unlikely to mediate the inhibitory effect of evoked exocytosis. Rather, SWCNTs could be inserting into/ plugging the fusion pore to produce such an effect. This decrease in astrocytic glutamate release in the presence of SWCNT-PEG could be beneficial in conditions like epilepsy where an increase in glutamate release from astrocytes has been linked to epileptic activity [14]. However, appropriate safety guidance and methods to deliver these nanomaterials into the brain parenchyma for therapeutic use are not defined at present.

The decrease in the total and vesicular recycling dye load that we observed in the presence of SWCNT-PEG (Figure 3) is qualitatively similar to that we previously observed in neurons [8]. As we used two different recycling dyes, FM4-64 here and FM1-43 in our previous work with neurons, known to have different properties [50], we stay away from temptation to make additional quantitative comparisons. However, it has to be stated as a well-accepted fact that the endocytosis/exocytosis process in astrocytes is much slower than that in neurons [24,25,40]. 

The process of constitutive exocytosis leads to the incorporation of new membrane and transporter-laden vesicles into the plasma membrane of astrocytes and this membrane can be retrieved by the process of endocytosis. Similar to many other membrane transporters, the glutamate transporters are also trafficked to and from the plasma membrane in an activity-dependent manner [51,52]. We show here that SWCNT-PEG blocks the process of constitutive endocytosis in astrocytes thereby causing an increase in the retention of new membrane leading to an increase in the area of astrocytes, as we reported previously [7,10,11]. This could also cause a decrease in the glutamate transporters being endocytosed once they are trafficked to the plasma membrane, hence causing an increase in the surface presence of the glutamate transporters and the resultant increase in glutamate uptake, as we have shown previously [11]. Though untested, this phenomenon could be explored in the future by transfecting the astrocytes with the plasmid encoding sodium-dependent excitatory amino acid transporters tagged with enhanced green fluorescence protein and record their vesicular trafficking [53] as well as plasma membrane dynamics while challenging astrocytes with SWCNT-PEG. 

The results on destaining recycling dye from astrocytes treated with SWCNT-PEG during second stimulus (Figure 4) are in stark contrast to the data obtained from neurons [8]. Such destaining was accelerated and more effective in neurons treated with SWCNT-PEG when compared to the untreated control neurons, both groups showing expected exponential rate of decay [8]. When this finding was combined with the reduction of endocytotic loading of the recycling dye during the first stimulation, we concluded that SWCNT-PEG preferentially inhibited regulated endocytosis [8]. Prior work showing that SWCNT-PEG dampens depolarization-induced cytoplasmic Ca^2+^ elevation in neurons [6] diminished a possibility that the faster rate of destaining was due to an increase in exocytosis. In the present work, destaining during the second stimulus was slower and less efficient in astrocytes treated with SWCNT-PEG than in untreated control astrocytes. In fact, this deficiency was so severe that the normal exponential rate of destaining decay seen in control astrocytes was not present in SWCNT-PEG-treated astrocytes and assumed a linear rate of decay (Figure 4). When these findings are combined with the reduction of endocytotic loading of the recycling dye during the first stimulation (Figure 3), we can only conclude that in astrocytes both arms of the regulated/evoked recycling pathway, endocytosis and exocytosis, are impaired by SWCNT-PEG. This gross difference between astrocytic and neuronal destainings could not be of technical nature, i.e., due to the use of different recycling dyes. The underlying molecular underpinnings of the dichotomy in destaining needs to be investigated, however. It is an attractive hypothesis that the difference in the average size of small/clear vesicles, ~40 nm in neurons [54,55] vs. ~300 nm in astrocytes [25,43] as well as in the microanatomy of vesicles and protein secretory machinery [13,25,41,43,55,56] might underlie the observed dichotomy in destaining. Another tempting speculation is that the nature of the stimulus, depolarization in neurons and ATP in astrocytes, could result in such a dichotomy. This notion is routed in our previous study showing that in astrocytes the proportion of two distinct types of events, transient and full fusions, was stimulus dependent, and so was the stability of the vesicle fusion pore [25].

Finally, we studied the effects of the PEG functional group on vesicular recycling in astrocytes. We previously demonstrated that the functional group PEG itself did not affect glutamate homeostasis unlike SWCNT-PEG that increased glutamate uptake (Figure 1B of ref. [11]) In the present work, PEG itself marginally inhibited constitutive endocytic load in astrocytes, but had no effect on ATP-evoked (pulse) endocytosis. Similarly, PEG also had some effect on the destaining curve during the second ATP stimulus (chase), showing slower and lesser destaining than the control. In all the conditions, PEG-treated groups showed a typical exponential decay of FM4-64 fluorescence. Taken together, we concluded that PEG itself does not mediate the major effects we observed that were exerted by SWCNT-PEG. However, data we obtained using PEG 600 (molecular weight range 573–630) might be considered unexpected. Namely, treatment of the drought-susceptible barrel clover (Medicago truncatula) accession Jemalong A17 for 5 days with 15% PEG (higher molecular weight of 8000) stimulated endocytosis in rhizodermal cells of the upper growth differentiation zone 4 of the roots. This effect, qualitatively opposite to what we observed in astrocytes using the same recycling dye FM4-64, was absent when the drought-resistant succession HM298 was used [57]. Undeniably, comparison between astrocytes isolated from the visual cortex of a rodent and rhizodermal cells in the roots of a legume may look awkward at the first sight. However, in legumes the PEG can simulate water deficit [57,58]. Given the well-recognized astrocytic function in water balance [59], it is tempting to speculate that the effects of PEG on vesicular recycling we observed in astrocytes may be related to the activity of aquaporin-4, a water channel expressed on the plasma membrane of astrocytes in the mammalian cerebrum [60].

## 5. Conclusions

**Figure 5:**
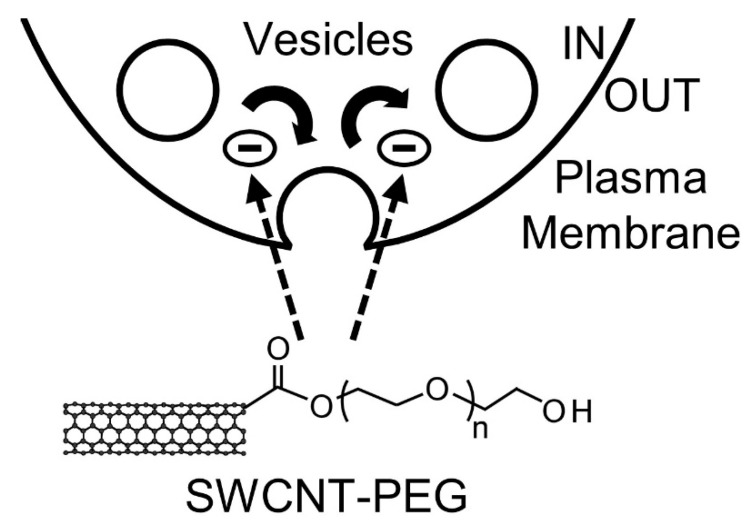
Scheme showing the possible mechanism of action of SWCNT-PEG solute on astrocytes. SWCNT-PEG obstructs vesicular/plasmalemmal recycling in astrocytes by inhibiting (**-**) both the endocytic and exocytotic pathways.

Here, we investigated the mechanism of action of SWCNT-PEG solute on astrocytes by studying their intracellular Ca^2+^ dynamics and the associated glutamate release along with their plasma membrane recycling. We show that the SWCNT-PEG solute does not affect the intracellular Ca^2+^ elevations in astrocytes but causes a reduction in the ATP-induced glutamate release. We also show that the SWCNT-PEG solute causes a decrease in the total and vesicular load of the recycling dye during ATP-stimulated recycling with obstructed release of the loaded recycling dye following ATP stimulation (Figure 5).

## Supplementary Materials

The following are available online at https://www.mdpi.com/2073-4409/9/7/1597/s1, Figure S1: CPA blocks the ATP-induced intracellular Ca^2+^ elevations in cultured mouse cortical astrocytes indicating that the ER Ca^2+^ store is the primary source of this increase in Ca^2+^, Figure S2: The decrease/trough in the fluorescence of iGluSnFR at the astrocytic plasma membrane observed after the addition of ATP is not caused by the plasma membrane dynamics as evidenced by the lack of change in the fluorescence of Lck_1-26_-EGFP, Figure S3: The decrease in the fluorescence of iGluSnFR observed after replacing the bath media is not ATP dependent, Figure S4: An example of rarely seen intercellular accumulation of SWCNT-PEG in an astrocyte.
