# Supplementary material for: Chemically Functionalized Water-Soluble Single-Walled Carbon Nanotubes Obstruct Vesicular/Plasmalemmal Recycling in Astrocytes Down-Stream of Calcium Ions
Source: Cells. 2020 Jul 1;9(7):1597. doi: 10.3390/cells9071597 (PMC7408470; doi:10.3390/cells9071597)
Supplement: Supplementary file 1 [file cells-09-01597-s001.pdf]

Supplementary material for:

Chemically functionalized water-soluble single-walled carbon nanotubes obstruct vesicular/plasmalemmal recycling in astrocytes down-stream of calcium ions

Manoj K Gottipati, Elena Bekyarova, Robert C Haddon, Vladimir Parpura

This file includes:

Supplementary methods

Supplementary figures S1-S4

References

Supplementary methods

We assessed the notion that the ATP-induced increase in intracellular calcium $[Ca^{2+}]_i$ in our cultured astrocytes is mainly via release from the endoplasmic reticulum (ER) Ca^{2+} store. To achieve this goal, we repeated the experiment described in Figure 1 with the addition of cyclopiazonic acid (CPA; 20 μM), a blocker of the ER store specific Ca^{2+}-ATPase responsible for (re)filling the store with Ca^{2+} for the cytosol. CPA was added to the external solution at the onset of the experiment and kept in the bathing media throughout the entire experiment. We found that CPA (n = 11) significantly blocked the increase in $[Ca^{2+}]_i$ (Figure S1A). We normalized the changes in $[Ca^{2+}]_i$ to their respective maximum $[Ca^{2+}]_i$ response following the application of 4-Br (Figure S1B) and found a significant reduction in the peak (by 85%) and cumulative (by 93%) normalized dF (Figure S1C), implying that the ER Ca^{2+} store is the primary source of this increase in $[Ca^{2+}]_i$ in our cultured astrocytes when stimulated with ATP.

Since iGluSnFR is anchored to the plasma membrane with the sensor on the extracellular side, we assessed if the trough observed following ATP stimulation might be an effect of plasma membrane dynamics or a transient dilution of the extracellular glutamate concentration. To accomplish that, we transfected astrocytes to express Lck_{1-26}-EGFP and repeated the experiment described in Figure 2. Lck_{1-26} sequence targets EGFP to the plasma membrane displaying EGFP on the extracellular side. We found that Lck_{1-26}-EGFP (n = 12), unlike iGluSnFR, did not show a trough in fluorescence following the replacement of external solution with ATP containing external solution (Figure S2) implying that the trough observed is not a result of plasma membrane dynamics.

It has been demonstrated that glutamate can induce the formation of filopodia in cultured hippocampal astrocytes [1,2]. Therefore, the application of glutamate to saturate iGluSnFR

fluorescence assessed the extent to which glutamate stimulation might have a direct effect on the plasma membrane dynamics in our cultured astrocytes. Consistent with our previous report [3], glutamate did not affect Lck_{1-26}-EGFP fluorescence dynamics. Possible reasons for discrepancies between work in cultured hippocampal [1,2] vs. our visual cortex astrocytes (here and elsewhere [3]) are that astrocytes originated from different brain regions and/or that astrocytes were grown on different strata as we discussed in our previous work [3].

Although iGluSnFR is a highly selective sensor for glutamate, it is possible that the trough could be a direct effect of ATP on the glutamate sensor. To assess this highly unlikely scenario, we repeated the experiment described in Figure 2, but the external solution was replaced with external solution not containing ATP in control and SWCNT-PEG treated iGluSnFR expressing astrocytes (Figure S3A). We normalized the changes in the extracellular glutamate levels to their respective maximum iGluSnFR fluorescence caused by extracellular glutamate application (100 μM) at the end of experiments, and found that the control cells (n = 6) as well as the CNT-treated cells (n = 6) showed a transient decrease in extracellular glutamate levels (Figure S3B) in the absence of ATP. We also found that the presence of SWCNT-PEG did not significantly affect this decrease in extracellular glutamate as evident by the lack of difference in the trough and cumulative normalized dF (Figure S3C). Taken together, the most plausible explanation for the observation of the trough in recoding or extracellular glutamate levels is a temporary decease in extracellular glutamate levels due to dilution imposed by bath application.

Supplementary figures

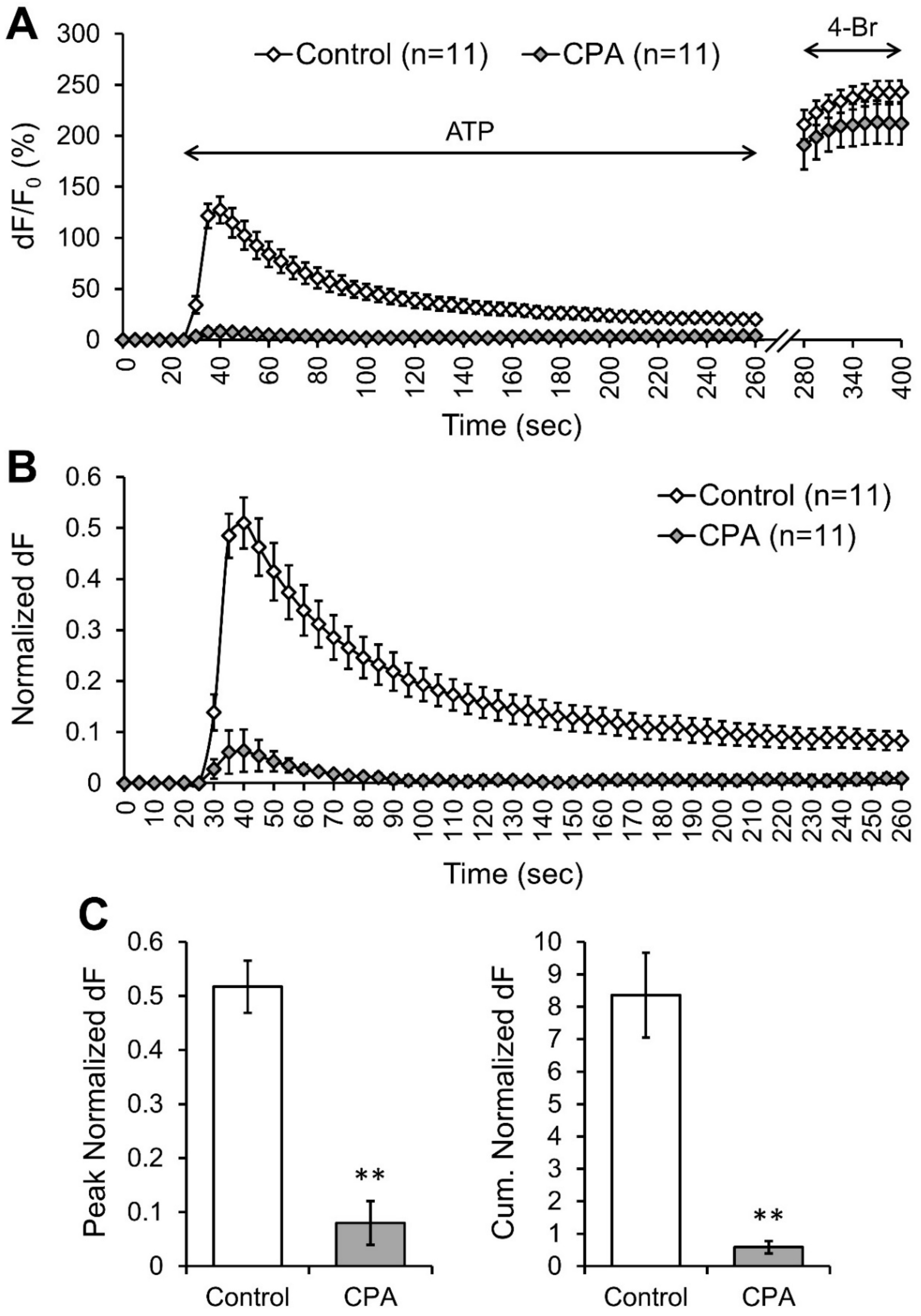

Figure S1. CPA blocks the ATP-induced intracellular Ca^{2+} elevations in cultured mouse cortical astrocytes indicating that the ER Ca^{2+} store is the primary source of this increase in Ca^{2+}. A) Time-lapse imaging of RCaMP1h fluorescence, reporting on the average intracellular Ca^{2+} levels in astrocytes in the absence and the presence of a blocker of ER Ca^{2+} ATPase, CPA (20 μM). ATP (100 μM) was bath applied to elicit an increase in intracellular calcium levels and 4-Bromo-A23187 (4-Br; 20 μM) was bath applied to elicit maximal Ca^{2+} response in astrocytes. To block the store-specific Ca^{2+} ATPase, CPA was applied to the bathing media at the onset of the experiment and kept in the bathing media throughout the entire experiment. The horizontal double-headed arrows indicate the times of addition of ATP and 4-Br containing external solutions. Changes in RCaMP1h fluorescence are expressed as dF/F_0 (%) after background subtraction. Number of astrocytes studied in each condition is shown in parentheses. The control group represents the control astrocytes reported in Figure 1 and replotted here, as these experiments were done in parallel. Thus, these experiments also represent a control for our ability to block ATP-induced Ca^{2+} dynamics in contrast to the inability of SWCNT-PEG to do so. B) Changes in the RCaMP1h fluorescence of astrocytes shown in A, normalized to their maximal Ca^{2+} response after the application of 4-Br, expressed as the normalized change in RCaMP1h fluorescence, dF. Traces in A and B show means ± SEMs. C) Summary graphs showing the average peak (left) and cumulative (right) normalized dF with SEMs. Asterisks indicate a statistical difference compared to the control. Student's t-test (pooled variances); $^{**}p < 0.01$.

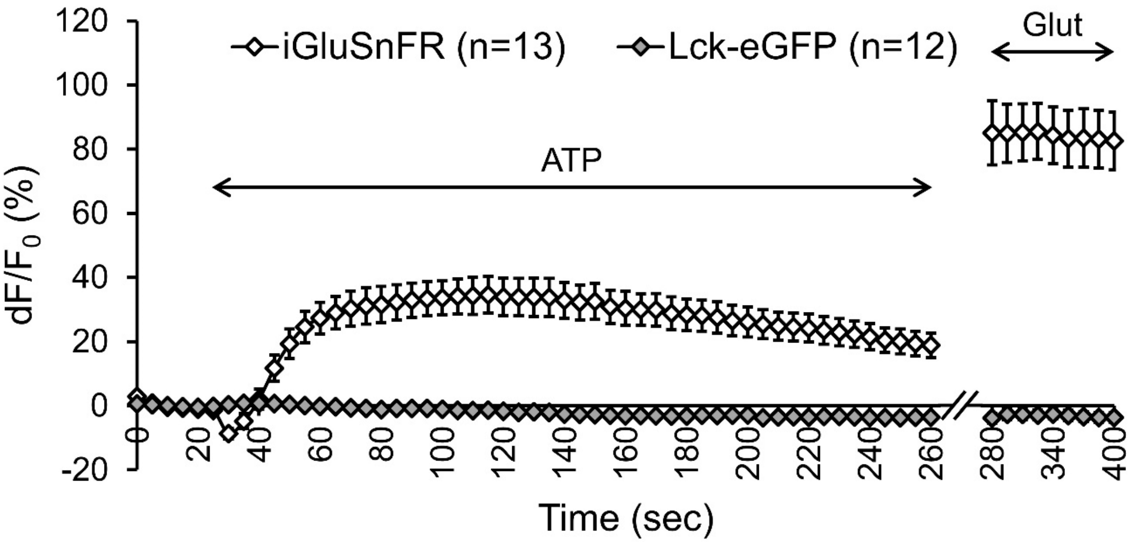

Figure S2. The decrease/trough in the fluorescence of iGluSnFR at the astrocytic plasma membrane observed after the addition of ATP is not caused by the plasma membrane dynamics as evidenced by the lack of change in the fluorescence of $Lck_{1\text{-}26}$-EGFP. Time-lapse imaging of iGluSnFR fluorescence, reporting on the average extracellular glutamate levels surrounding astrocytes along with the time-lapse imaging of $Lck_{1\text{-}26}$-EGFP fluorescence reporting on the plasma membrane dynamics. ATP (100 μM) was bath applied to stimulate glutamate release from astrocytes and glutamate (Glut; 100 μM) was bath applied to saturate the iGluSnFR fluorescence in astrocytes. Note that glutamate does not affect $Lck_{1\text{-}26}$-EGFP fluorescence (see supporting information text for details). The horizontal double-headed arrows indicate the times of addition of ATP and glutamate containing external solutions. Changes in iGluSnFR and $Lck_{1\text{-}26}$-EGFP fluorescence are expressed as dF/F_0 (%) after background subtraction. Number of astrocytes studied in each condition is shown in parentheses. The control group represents the control astrocytes reported in Figure 2 and replotted here, as these experiments were done in parallel.

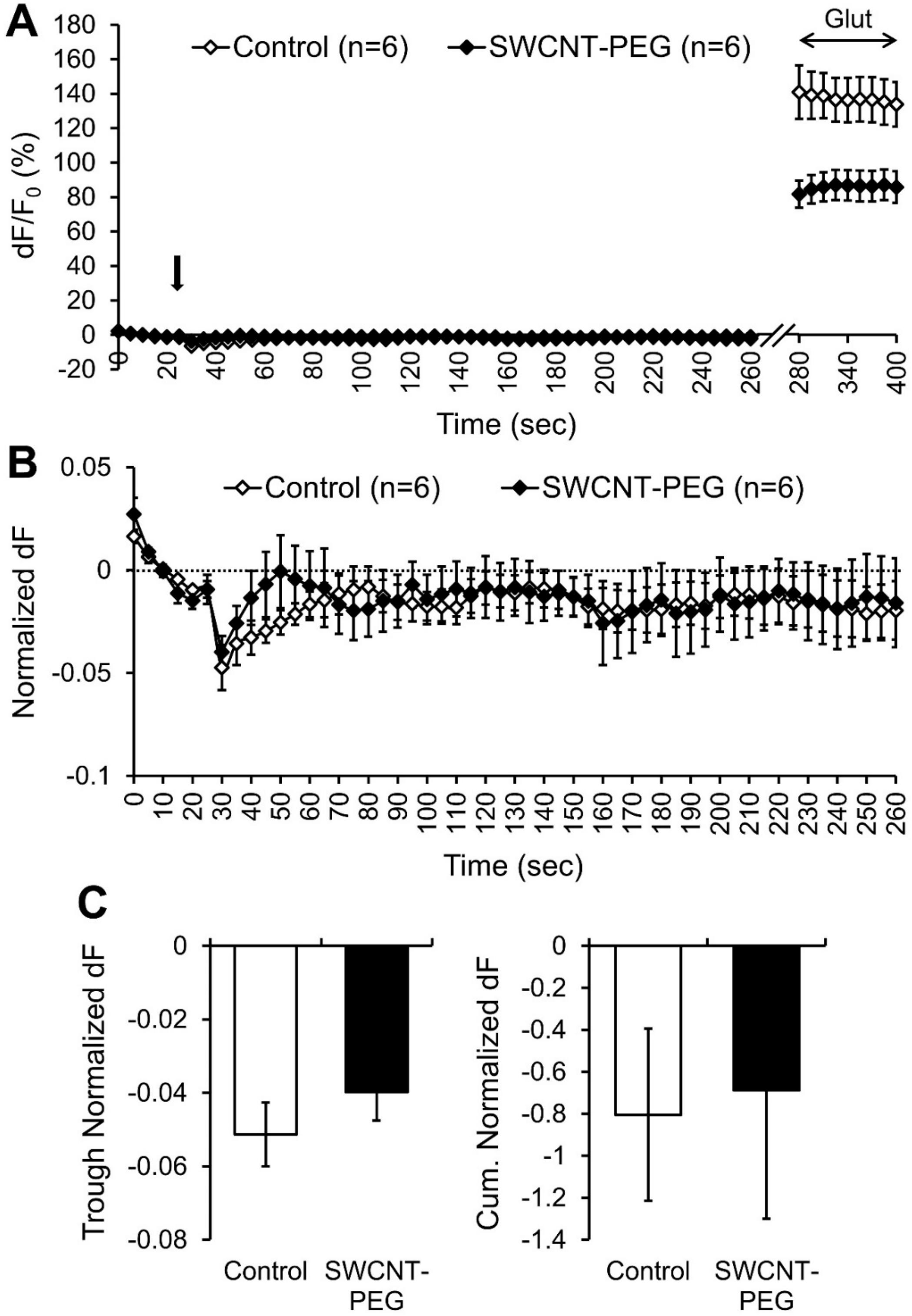

Figure S3. The decrease in the fluorescence of iGluSnFR observed after replacing the bath media is not ATP dependent. A) Time-lapse imaging of iGluSnFR fluorescence, reporting on the

average extracellular glutamate levels in astrocytes in the absence and the presence of SWCNT-PEG solute (5 μg/ml). The arrow indicates the time of replacing the bath media and the horizontal double-headed arrow indicates the time of addition of glutamate (Glut; 100 μM) containing external solution to saturate the iGluSnFR fluorescence in astrocytes. Changes in iGluSnFR fluorescence are expressed as dF/F_0 (%) after background subtraction. Number of astrocytes studied in each condition is shown in parentheses. B) Changes in the iGluSnFR fluorescence of astrocytes shown in A, normalized to their saturated glutamate response after the application of glutamate, expressed as the normalized change in iGluSnFR fluorescence, dF. Traces in A and B show means ± SEMs. C) Summary graphs showing the average trough (left) and cumulative (right) normalized dF with SEMs.

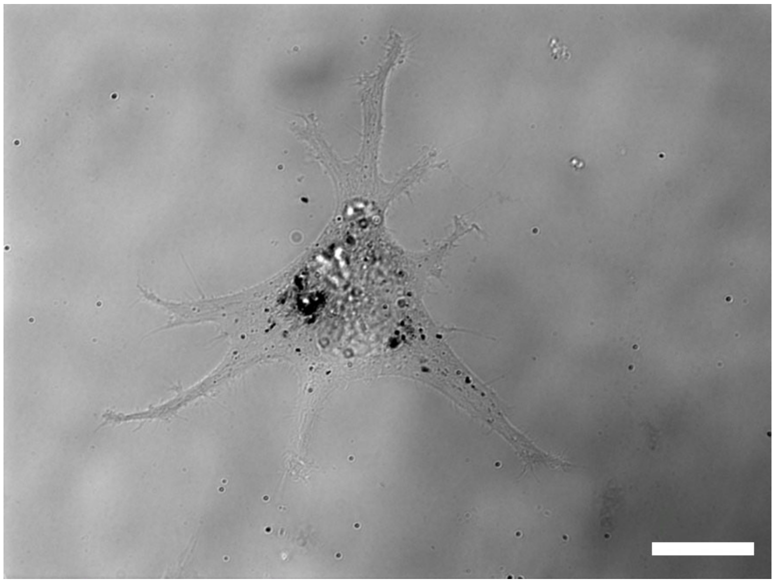

Figure S4. An example of rarely seen intercellular accumulation of SWCNT-PEG in an astrocyte. Bright filed image of a control astrocyte incubated for 4 days with SWCNT-PEG (5 μg/ml). Black dots in the cytoplasm of the astrocyte represent the accumulation of SWCNT-PEG. Scale bar, 20 μm.

References

1. Cornell-Bell, A.H.; Thomas, P.G.; Smith, S.J. The excitatory neurotransmitter glutamate causes filopodia formation in cultured hippocampal astrocytes. *Glia* **1990**, *3*, 322-334, doi:10.1002/glia.440030503.

2. Lavialle, M.; Aumann, G.; Anlauf, E.; Prols, F.; Arpin, M.; Derouiche, A. Structural plasticity of perisynaptic astrocyte processes involves ezrin and metabotropic glutamate receptors. *Proc Natl Acad Sci U S A* **2011**, *108*, 12915-12919, doi:10.1073/pnas.1100957108.

3. Lee, W.; Parpura, V. Spatio-temporal characteristics of metabotropic glutamate receptor 5 traffic at or near the plasma membrane in astrocytes. *Glia* **2016**, *64*, 1050-1065, doi:10.1002/glia.22982.
